# Circulating Kidney Injury Molecule 1 Predicts Prognosis and Poor Outcome in Patients With Acetaminophen‐Induced Liver Injury

**DOI:** 10.1002/hep.27857

**Published:** 2015-05-29

**Authors:** Daniel J. Antoine, Venkata S. Sabbisetti, Ben Francis, Andrea L. Jorgensen, Darren G.N. Craig, Kenneth J. Simpson, Joseph V. Bonventre, B. Kevin Park, James W. Dear

**Affiliations:** ^1^ MRC Center for Drug Safety Science, Department of Molecular & Clinical PharmacologyInstitute of Translational MedicineUniversity of LiverpoolLiverpoolUnited Kingdom; ^2^ Renal Division, Department of MedicineBrigham and Women's Hospital, Harvard Medical SchoolBostonMA; ^3^ Department of BiostatisticsInstitute of Translational MedicineUniversity of LiverpoolLiverpoolUnited Kingdom; ^4^ Scottish Liver Transplantation UnitRoyal Infirmary of EdinburghEdinburghUnited Kingdom; ^5^ Pharmacology, Toxicology and TherapeuticsUniversity/BHF Center for Cardiovascular ScienceEdinburgh University & NPIS Edinburgh, Scottish Poisons Information BureauEdinburghUnited Kingdom.

## Abstract

Acute kidney injury in the context of acetaminophen (APAP; paracetamol)‐induced liver injury is an important predictor of the requirement for urgent liver transplantation (LT) to avoid death. However, the prognostic biomarker used to report kidney dysfunction (serum creatinine concentration) has suboptimal sensitivity and specificity. Kidney injury molecule 1 (KIM‐1) can be quantified in plasma as a sensitive and specific biomarker of kidney injury in both clinical and preclinical studies. Therefore, plasma KIM‐1 has potential as a sensitive prognostic biomarker of patient outcome post‐APAP overdose. In a cohort of APAP overdose patients (N = 74) with and without established liver injury, we quantified plasma KIM‐1 by immunoassay on the first day of admission to a LT unit and assessed its diagnostic performance to predict outcome compared with serum creatinine concentration. Day 1 plasma KIM‐1 was significantly elevated in patients that died or required LT, compared to spontaneous survivors (1,182 ± 251 vs. 214 ± 45 pg/mL; *P* < 0.005). Receiver operator characteristic analysis demonstrated the superiority of KIM‐1 (area under the curve [AUC]: 0.87; 95% confidence interval [CI]: 0.78‐0.95; 0.56 sensitivity at 0.95 specificity), compared with serum creatinine (AUC, 0.76; 95% CI: 0.64‐0.87; 0.08 sensitivity at 0.95 specificity) and other current prognostic indicators, when measured on the first day of enrollment into the study. Furthermore, KIM‐1 was found to be a statistically significant independent predictor of outcome at the 5% level (*P* < 0.0386) in a multivariable logistic regression model that considered all measured factors (pseudo‐R^2 = 0.895). *Conclusion*: Early measurement of plasma KIM‐1 represents a more sensitive predictor of patient outcome than serum creatinine concentration post‐APAP overdose. With further development, plasma KIM‐1 could significantly improve prognostic stratification. (Hepatology 2015;62:591–599

**Abbreviations**
AKI: acute kidney injury
ALF: acute liver failure
ALI: acute liver injury
ALT: alanine aminotransferase
APAP: acetaminophen
AUC: area under the curve
AUC‐ROC: area under the receiver operator characteristic curve
CI: confidence interval
CKD: chronic kidney disease
HMGB1: high‐mobility group box 1
IV: intravenous
KCC: King's College Criteria
KIM_1: kidney injury molecule 1
LT: liver transplantation
MELD: Model for End‐Stage Live Disease
NAC: *N‐*acetylcysteine
PT: prothrombin time
ROC: receiver operator characteristic
SD: standard deviation
SEM: standard error of the mean
SLTU: Scottish Liver Transplantation Unit
SOFA: Sequential Organ Failure Assessment

Acetaminophen (APAP; paracetamol) is the most common cause of acute liver failure (ALF) in the Western world and is responsible for approximately 200 and 500 deaths per year in Britain and the United States, respectively.^1^, ^2^ The translational multimechanistic events of APAP liver toxicity are well described^3^ and can be monitored with circulating mechanism‐based biomarkers in human and murine models.^4^, ^5^, ^6^, ^7^ However, APAP can also induce kidney tubular cell death, resulting in acute kidney injury (AKI).^8^ In the presence of APAP‐induced acute liver injury (APAP‐ALI), AKI is one of the key predictors of death and requirement for urgent liver transplantation (LT), with kidney function being a key component of the King's College Criteria (KCC)^9^ and the Model for End‐Stage Liver Disease (MELD)^10^ scoring systems that are used for prognostic stratification. Moreover, it has been shown that patients with both APAP and non‐APAP ALF that develop AKI have a significantly prolonged hospital admission and reduced spontaneous survival, compared with those that do not develop AKI.^11^

Kidney function is determined by serum creatinine concentration that is used in KCC and MELD. However, patients with AKI are not in steady state with regard to kidney function, and serum creatinine is slow to report AKI. Serum creatinine also lacks specificity, becoming elevated by nonrenal pathologies, such as dehydration and muscle injury.^12^ The kinetic limitations of serum creatinine significantly reduce the early prognostic accuracy of KCC and MELD.

Substantial research efforts have been aimed at the discovery and qualification of new biomarkers of kidney and liver toxicity.^13^ Most of the biomarkers for renal injury and dysfunction have been described in urine and hence require a sample to be available at the time of making acute clinical decisions. The wide variation in urine concentration across and within patients complicates biomarker interpretation, with normalization being required. When patients are anuric, urinary biomarkers cannot evaluate the extent or localization of injury or provide mechanistic information that may inform treatment options. Blood‐based biomarkers have the potential to overcome these limitations^14^ and have the advantage over urinary‐based markers with respect to on‐demand access at the time of acute clinical decision making. In the setting of APAP toxicity, earlier, more specific reporting of renal dysfunction could improve prognostic accuracy, accelerate appropriate patient listing for LT and guide entry into clinical trials of novel therapeutic approaches.

Kidney injury molecule 1 (KIM‐1) is a transmembrane glycoprotein that is highly expressed in kidney proximal tubular cells with kidney injury.^15^ The KIM‐1 ectodomain is cleaved, released into urine, and can be quantified as a sensitive and specific biomarker for AKI in rodents^16^ and humans.^17^ KIM‐1 reports AKI secondary to a wide range of toxic, ischemic, and septic insults. Urinary KIM‐1 has undergone formal qualification review and has been qualified by regulatory agencies to be used to support preclinical drug discovery programs.^18^ In relation to liver disease, urinary KIM‐1 is elevated in patients with cirrhosis with a clinical diagnosis of acute tubular necrosis more than in those with hepatorenal syndrome and may, with development, have utility as a diagnostic biomarker.^19^ Recently, Sabbisetti et al. demonstrated that KIM‐1 is released into the circulation where it reports kidney injury in mice, rats, and humans with AKI and chronic kidney disease (CKD).^20^ In this current investigation, we build on the work reported by Sabbisetti et al. and investigate the hypothesis that measurement of circulating KIM‐1 during APAP hepatotoxicity can represent a more sensitive and specific predictor of patient outcome than serum creatinine.

## Patients and Methods

#### APAP‐ALI Patients

The study was prospectively approved by the local research ethics committee and informed consent was obtained from all patients, or the patient's next of kin, before entry into the study. A total of 67 adult (age >16 years) patients transferred to the Scottish Liver Transplantation Unit (SLTU), Royal Infirmary of Edinburgh (Edinburgh, UK) with ALI, secondary to APAP ingestion (single overdose, staggered overdose, or therapeutic excess), were entered into the study (day 1 is the day of admission to SLTU). ALI was defined as an increase in serum alanine aminotransferase (ALT) activity above 3× upper limit of normal with a sudden deterioration in liver function with associated coagulopathy in the absence of a history of chronic liver disease with a clear history of excess APAP ingestion. Clinical parameters recorded for each patient during hospitalization included age, gender, encephalopathy grade, and outcome (survived, died, or underwent LT). Laboratory parameters measured daily for each patient included ALT activity, serum creatinine concentration, prothrombin time (PT), and full blood count. Illness severity was quantified daily by KCC and the Sequential Organ Failure Assessment (SOFA) score.^21^ All patients received intravenous (IV) *N*‐acetylcysteine (NAC) treatment.

#### APAP Overdose Without Organ Injury (APAP‐No‐ALI Patients)

Seven patients were recruited from the Royal Infirmary of Edinburgh. Consent was obtained from all patients before recruitment, and the study received appropriate local ethical approval. The entry criterion was a history of a single APAP ingestion in overdose that required treatment with NAC as per UK guidelines at time of hospital admission. All patients had a serum APAP concentration above the “200” line on the treatment nomogram. Blood was collected at the end of IV NAC infusion for measurement of serum ALT activity and other biomarkers. Absence of liver injury was confirmed by a normal serum ALT activity (<50 U/L).

In both APAP‐ALI and APAP‐no‐ALI patient groups, peripheral blood samples were collected and immediately centrifuged at 1,000*g* for 15 minutes at 4^0^C. The supernatant was then separated into aliquots and stored at −80^0^C until analysis.

#### Plasma KIM‐1 Quantification

Plasma KIM‐1 concentrations were measured using microsphere‐based Luminex technology, as previously described for human plasma.^20^ Analytes were quantified using a 13‐point five parametric logarithmic standard curve. Inter‐ and intra‐assay variability was less than 15%. Investigators performed all KIM‐1 measurements blindly and were unaware of patients’ clinical characteristics. Mean (95% confidence interval [CI]) plasma KIM‐1 concentration in healthy volunteers has been reported as 64.4 (51.0‐77.7) pg/mL.

#### Statistical Analysis

Data are presented as mean ± standard error of the mean (SEM). Each data set was analyzed for non‐normality using Shapiro‐Wilk's test. For two non‐normal data sets, comparisons were made using Mann‐Whitney's U test. Kruskall‐Wallis’ test was used to determine significance between more than two non‐normal sample groups. All calculations were performed using StatsDirect statistical software (StatsDirect Ltd., Altrincham, UK). For correlative analysis, Pearson's correlation test, *R* ^2^, and receiver operator characteristic (ROC) area under the curve (AUC) analysis were carried out using GraphPad PRISM software (Graphpad Software Inc., La Jolla, CA). Results were considered significant when *P* < 0.05. Logistic regression analysis was performed using R software (R Foundation for Statistical Computing, Vienna, Austria), with the statistical significance of covariates assessed using Wald's test. To estimate the total variability in outcome explained by the fitted model, its pseudo‐R^2 value was calculated. The funding sources had no influence over the study design, data analysis, or manuscript production.

## Results

#### Characteristics of the Patient Study Cohort

**Figure 1:**
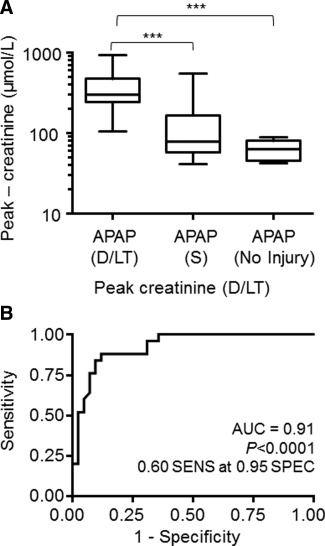
Peak serum creatinine concentration predicts patient outcome post‐APAP overdose. (A) Relationship between peak serum creatinine concentration and outcome (D/LT, death/liver transplant; S, spontaneous survival; No injury, no liver or renal toxicity) post‐APAP overdose. Peak serum creatinine occurred at 2.5 ± 1.1 (mean ± SD) days postadmission to the tertiary LT unit. Data are represented as box plots with maximum and minimum values indicated. Statistical significance was assigned as: *P < 0.05; **P < 0.01; ***P < 0.005. (B) ROC curve analysis for prediction of patient outcome (death/LT) by peak serum creatinine. AUC and sensitivity at 0.95 specificity are given with statistical significance. Abbreviations: SENS, sensitivity; SPEC, specificity.

Table 1 summarizes patient demographics as well as laboratory clinical chemistry parameters measured to assess liver and kidney function on day 1 of enrollment to this single‐center study. In total, 74 patients were recruited to the study. From this cohort of patients, 7 did not develop liver or kidney injury. Of the patients that developed ALI (N = 67), 42 spontaneously survived and 25 either died (N = 16) or received LT (N = 9). Within these subgroups of patients, serum ALT activity was not significantly different (Table 1). However, PT was significantly elevated in the group that required LT/died (67.2 ± 9.1 seconds), compared to the group that spontaneously survived (43.8 ± 4.0 seconds; Table 1). Peak hospital‐stay serum creatinine concentration represents a strong predictor of patient outcome and requirement of LT postoverdose, and this was reflected in our patient cohort with ALI (Table 1; Fig. 1) and supported by AUC‐ROC (area under the curve/receiver operator characteristic) curve analysis (AUC‐ROC, 0.92; 95% CI: 0.86‐0.97). However, peak serum creatinine concentration was delayed until 2.5 ± 1.1 (mean ± standard deviation [SD]) days postenrollment into the study.

**Table 1:** Patient Demographics and Biomarker Characteristics Measured Post‐APAP Overdose

	Outcome		
	LT/Died	Survive	Survive—No Liver or Renal Injury
No.	25	42	7
Age, years	46 ± 16	37 ± 12	33 ± 9
Day 1 serum ALT, U/L	4,342.7 ± 784.4	5,199.7 ± 530.3	30.7 ± 6.9***
Day 1 PT time, seconds	67.2 ± 9.1*	43.8 ± 4.0	N/D
Day 1 serum creatinine, µmol/L	213.8 ± 22.6*	120.8 ± 15.2	62.0 ± 17.8
Peak serum creatinine, µmol/L	379.8 ± 41.9***	139.3 ± 21.2	78.7 ± 10.2
Day 1 plasma KIM‐1, pg/mL	1,182.2 ± 251.4***	214.3 ± 45.3	112.7 ± 20.9

Comparison of the standard clinical chemistry parameters used to assess liver (ALT and PT) and renal function (creatinine) with novel biomarkers (KIM‐1) from patients that survive, die/require LT, or survive without liver or kidney damage post‐APAP overdose. Biomarker determinations were calculated from a blood sample obtained on day 1 of admission to the tertiary LT unit. Data are represented as mean ± SEM. Statistical significance was assigned as *P < 0.05 and ***P < 0.005 for either overdose patients that died/required LT or surviving patient without liver or renal injury compared to surviving overdose patients.

Abbreviation: N/D, not determined.

#### Relationship Between Day 1 Circulating KIM‐1 and Creatinine With Patient Outcome

**Figure 2:**
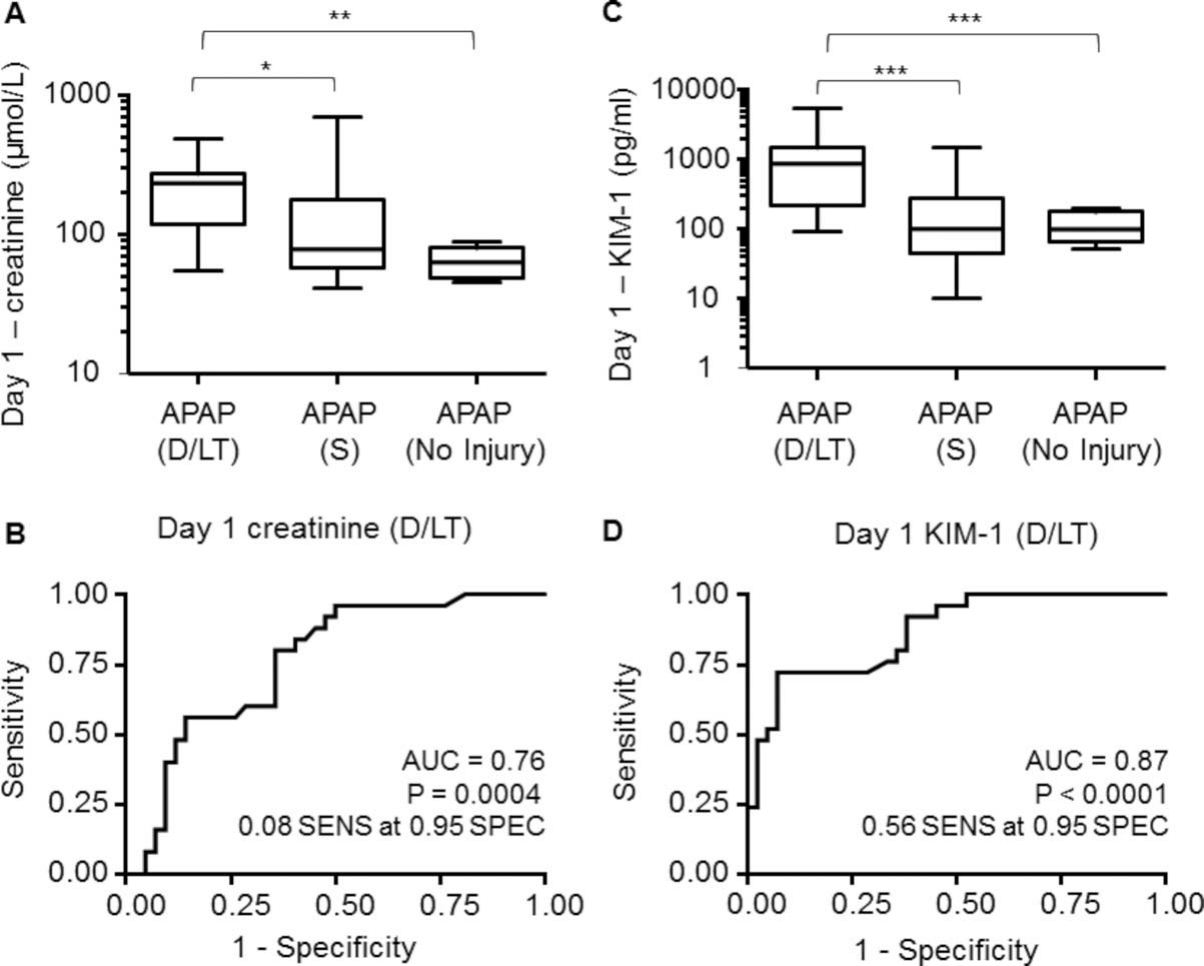
Plasma KIM‐1 outperforms serum creatinine at early time points for prediction of patient outcome post‐APAP overdose. (A and B) Relationship between serum creatinine concentration or (C and D) plasma KIM‐1 and outcome (D/LT, death/LT; S, spontaneous survival; No injury, no liver or renal toxicity) post‐APAP overdose. Serum creatinine and plasma KIM‐1 were determined on day 1 of admission to the tertiary LT unit. (A and C) Data are represented as box plots with maximum and minimum values indicated. Statistical significance was assigned as: *P < 0.05; **P < 0.01; ***P < 0.005. (B and D) ROC curve analysis for prediction of patient outcome (death/LT) by either serum creatinine or plasma KIM‐1. AUC and sensitivity at 0.95 specificity are given with statistical significance. Abbreviations: SENS, sensitivity; SPEC, specificity.

**Figure 3:**
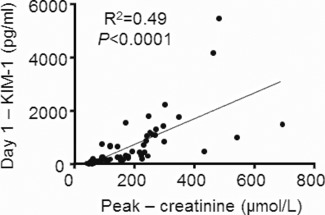
Relationship between day 1 plasma KIM‐1 and peak serum creatinine. Correlation of plasma KIM‐1 measured on day 1 of admittance into the study with peak serum creatinine. Peak serum creatinine occurred at 2.5 ± 1.1 (mean ± SD) days postadmission to the tertiary LT unit enrolment. *R* ^2^ value given with statistical significance. N = 74 patients.

Day 1 serum creatinine was significantly elevated in patients that reached our primary endpoint of either died or required LT (213.8 ± 22.6 µmol/L), compared to spontaneous survivors (120.8 ± 15.2 µmol/L; Table 1; Fig. 2) with an AUC‐ROC of 0.76 (95% CI: 0.64‐0.87) and sensitivity of 0.08 at 0.95 specificity (Fig. 2). In comparison with creatinine, plasma KIM‐1 on day 1 was increased with a higher mean fold change (5.5 vs. 1.8, KIM‐1 and creatinine, respectively) in patients that died or required LT, compared to spontaneous survivors. The AUC‐ROC for predicting outcome based upon day 1 plasma KIM‐1 concentration was 0.87 (95% CI: 0.78‐0.95), and sensitivity was 0.56 for plasma KIM‐1 concentration with specificity of 0.95 (Fig. 2). The AUC‐ROC and sensitivity at 0.95 specificity of day 1 plasma KIM‐1 was similar to peak serum creatinine, and there was a strong and significant correlation (*R* ^2^ = 0.49; *P* < 0.0001) between these two measurements (Fig. 3). Table 2 compares day 1 plasma KIM‐1 with other biomarkers of liver injury/prognosis with regard to our primary endpoint of death or receiving LT.

**Table 2:** Comparison of Liver Injury or Function Biomarkers at Early Time Points for the Prediction of Patient Outcome Post‐APAP Overdose

Biomarker (day 1)	AUC‐ROC	95% CI	Sens at 0.95 Spec
KIM‐1	0.87	0.78‐0.95	0.56
SOFA score	0.81	0.71‐0.86	0.55
Encephalopathy grade	0.77	0.64‐0.90	0.52
MELD	0.77	0.66‐0.89	0.14
Creatinine	0.76	0.64‐0.87	0.08
Total bilirubin	0.65	0.51‐0.78	0.42
PT	0.62	0.48‐0.77	0.23
ALT	0.59	0.44‐0.74	0.06

Biomarkers were measured on day 1 of admission to the tertiary LT unit. AUC‐ROC analysis is given for prediction of outcome on study day 1 with 95% CI and the recording of sensitivity at 0.95 specificity (Sens at 0.95 Spec). Day 1 is defined as day of admission to the tertiary LT unit.

#### Relationship Between Day 1 Circulating KIM‐1 and Creatinine With Patient Prognosis

**Figure 4:**
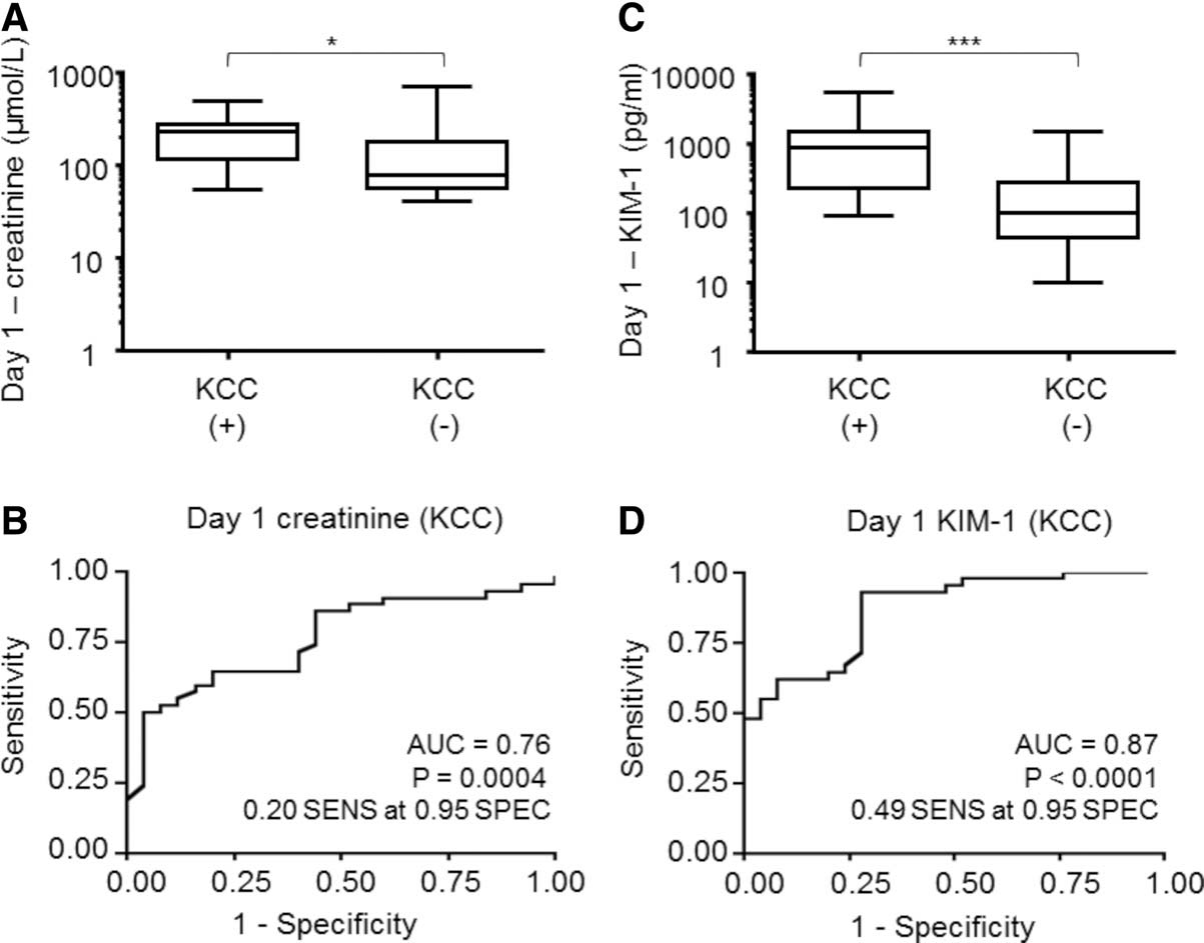
Plasma KIM‐1 outperforms serum creatinine at early time points for prediction of patients reaching KCC. (A and B) Relationship between serum creatinine concentration or (C and D) plasma KIM‐1 and outcome (reaching KCC [+] or not [−]) post‐APAP overdose. Serum creatinine and plasma KIM‐1 were determined on day 1 of admission to the tertiary LT unit. (A and C) Data are represented as box plots with maximum and minimum values indicated. Statistical significance was assigned as: *P = 0.03; ***P < 0.0001. (B and D) ROC curve analysis for prediction of patient outcome (KCC+) by either serum creatinine or plasma KIM‐1. AUC and sensitivity at 0.95 specificity are given with statistical significance. Abbreviations: SENS, sensitivity; SPEC, specificity.

**Figure 5:**
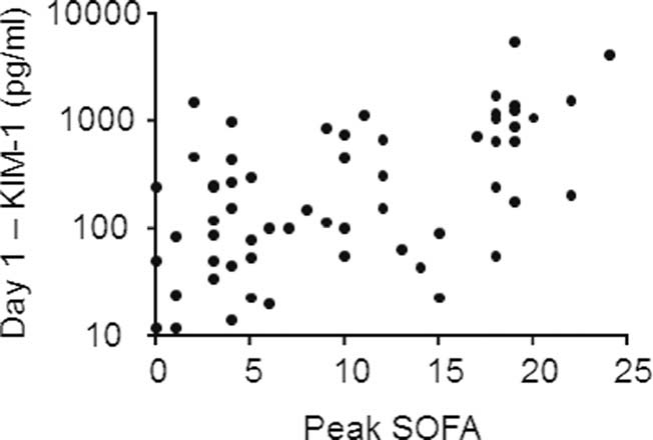
Relationship between day 1 plasma KIM‐1 and peak SOFA score. Correlation of plasma KIM‐1 measured on day 1 of admission to the tertiary LT unit with peak SOFA score.

The relationship between day 1 plasma KIM‐1 and current prognostic tools was also explored. Despite serum creatinine being a central component of KCC, KIM‐1 predicted reaching KCC with a larger ROC‐AUC, compared with day 1 serum creatinine (KIM‐1: AUC, 0.87; 95% CI: 0.78‐0.95; 0.49 sensitivity at 0.95 specificity; creatinine: AUC, 0.76; 95% CI: 0.64‐0.88; 0.20 sensitivity at 0.95 specificity; Fig. 4). There was also a statistically significant correlation between day 1 plasma KIM‐1 and hospital‐stay peak SOFA score (*R* ^2^ = 0.24; *P* < 0.0001), although the correlation was weaker compared with the correlation with peak serum creatinine alone (Fig. 5).

#### Relationship Between Day 1 Circulating KIM‐1 and Patient Outcome Once Other Variables Are Accounted For

The association between circulating KIM‐1 concentration, when measured on day 1 of study admittance, and patient outcome post‐APAP overdose after other important variables had been accounted for was determined by using a logistic regression model. Covariates of gender, SOFA, ALT, encephalopathy grade, PT, creatinine, and bilirubin were included in a logistic regression model together with our variable of interest, KIM‐1. Day 1 circulating KIM‐1 concentration was found to remain statistically significant at the 5% level (*P* = 0.0386), even when all other covariates were adjusted for, and the fitted model accounted for approximately 89.5% variability in patient response (pseudo‐R^2 = 0.895; Table 3).

**Table 3:** Determination of Significant Independent Predictors of Patient Outcome by Multivariable Logistic Regression Analysis

Factor	Coefficient	Standard Error	*P* Value
Intercept	−8.52	4.65	0.067
Gender	−4.61	2.51	0.066
SOFA	1.24	0.65	0.054
ALT	0.00018	0.00016	0.270
Encephalopathy grade			0.470
1	2.17	2.21	
2	6.24	3.59	
3	−1.18	3.16	
4	0.99	4.75	
PT	0.043	0.031	0.160
Creatinine	−0.046	0.025	0.060
Bilirubin	0.0072	0.010	0.490
KIM‐1	0.011	0.0051	0.039*

Biomarkers and clinical assessments were measured on day 1 of admission to the tertiary LT unit. Day 1 is defined as day of admission to the tertiary LT unit. Factors explored individually in Table 2 were combined into a multivariable logistic regression model. Overdose patients that died/required LT were coded as a “1” outcome and surviving patient without liver or renal injury were coded as a “0” outcome. Logistic regression coefficients, standard errors, and *P* values are displayed for each of the factors. Statistical significance was assigned as *P < 0.05.

## Discussion

Kidney function is a key predictor of outcome in patients with APAP‐ALI.^22^ Currently, renal function and prognostication are based on determination of serum creatinine concentration, which is relatively nonspecific and insensitive. Therefore, there is an urgent need to identify and validate biomarkers that show both improved sensitivity and specificity to assist clinical management of APAP poisoning. This study has demonstrated, for the first time, that elevations in a new sensitive and specific kidney injury biomarker—plasma KIM‐1—can predict patient outcome during APAP poisoning. Our data also provide evidence that circulating KIM‐1 may outperform other circulating markers of renal toxicity used for prognostic determination, such as serum creatinine concentration. Furthermore, in a logistic regression model of multivariable analysis, circulating KIM‐1 remained a significant independent predictor of outcome when measured on day 1 of study entry. With further qualification, this biomarker could refine patient stratification and improve LT decisions.

KIM‐1 is highly expressed postinjury to the kidney's proximal tubule and has been qualified as a biomarker of AKI in urine for preclinical investigations.^18^, ^23^ Recently, circulating KIM‐1 has been reported to be increased in rodent models of, and humans with, AKI and CKD, but not elevated in murine models of chemical‐induced liver injury.^20^ In mice, circulating KIM‐1 concentration tracks the increase in kidney expression, and in humans, plasma and urinary KIM‐1 are positively correlated. The present study builds on this and is the first to demonstrate that KIM‐1 is more sensitive and accurate than serum creatinine concentration with regard to reporting outcome after human APAP overdose. Although clinical adjudication represents the best possibility of accurately phenotyping patients, the true gold standard to assess degree of renal injury would be through biopsy. Without tissue histology to quantify the actual degree of clinical tubular damage in these patients, the observed quantification of kidney injury by plasma KIM‐1 may or may not exaggerate the real rate of tubular injury. Renal biopsy represents a significant risk to these patients, so development of novel biomarkers is imperative. However, it is important to note that the lack of human tissue to anchor biomarker values against represents a common challenge for translational biomarker qualification and research. Moreover, the data presented in this article are consistent with those obtained from preclinical investigations were circulating levels of KIM‐1 could be correlated with the actual degree of renal injury.^19^

Serum creatinine represents a central component of the KCC for APAP overdose. Despite this, day 1 plasma KIM‐1 predicted reaching KCC with greater superiority, compared with day 1 serum creatinine. The superior diagnostic performance of plasma KIM‐1 in this investigation was demonstrated by larger AUC‐ROC curves (compared with creatinine) for the endpoints of death/LT and reaching KCC as well as in a logistical regression model when all other covariates were adjusted for. Furthermore, day 1 plasma KIM‐1 was also superior to encephalopathy grade, bilirubin concentration, PT, and serum ALT for prediction of our primary endpoint. Though this is consistent with KIM‐1 having potential as a clinically useful biomarker, these data should be interpreted as hypothesis generating. Our patient cohort was recruited on admission to an LT unit and is liable to selection bias. For example, patients will have been selected for admission based on established prognostic markers, such as PT, and this will tend to minimize between‐group differences in conventional biomarkers. In clinical practice, a biomarker cut‐off value is often used to distinguish normal from disease. A cut‐off of 300‐µmol/L serum creatinine concentration is used in KCC. When the cut‐off biomarker value at 0.95 specificity was compared, the difference in sensitivity between KIM‐1 and creatinine was substantial (0.56 vs. 0.08, respectively). In the context of prognostic stratification of patients with APAP‐ALI, there is a need to maximize both sensitivity and specificity—patients with a high risk of death must be correctly identified early to maximize their chance of life‐saving transplantation. However, transplantation will do more harm than good if patients are misclassified as needing transplantation when spontaneous recovery would otherwise occur.^24^ The improved sensitivity and specificity of plasma KIM‐1 have potential to improve decisions regarding patient transfer to specialist LT centers and listing for transplantation. One strategy to move further forward to understand the clinical utility and interpretation of plasma KIM‐1 in the context of APAP overdose is to define normal reference ranges and establish cut‐off values in healthy population and populations with renal disease and evaluate whether there is any impact of gender, ethnicity, and diurnal variation on plasma KIM‐1 expression levels.

The findings in this current investigation and our conclusions are based on the retrospective measurement of plasma KIM‐1 in a single‐center cohort of a relatively low number of patients at a single time point and are hypothesis generating. Future development requires a larger, validation‐prospective, multicenter assessment as well as a detailed kinetic analysis of the concentration of plasma KIM‐1 in relation to time postoverdose, time to treatment with NAC, and assessment against plasma APAP concentration. The patient groupings utilized within our investigation already had established ALI and had been transferred to a specialist treatment center. Further investigations are required to determine the diagnostic potential of plasma KIM‐1 to predict patient outcome at the hospital front door or as soon as possible postadmission to a general hospital emergency department. However, the data presented within this investigation, for the first time, support the superiority of plasma KIM‐1 versus serum creatinine, SOFA, or MELD with respect to sensitivity and specificity to predict patient prognosis and outcome during APAP hepatotoxicity. Moreover, although only investigated in a small subgroup of patients with APAP overdose that did not develop organ injury, we have shown that APAP exposure does not elevate plasma KIM‐1 above values observed in healthy volunteers.

A number of new biomarkers have recently been identified that promise to refine the management of APAP overdose. They have high liver specificity (e.g., microRNA‐122^25^) or provide a readout of the active mechanisms of cellular injury (e.g., high‐mobility group box 1 [HMGB1],^5^ keratin‐18,^5^ and mitochondrial DNA^6^). At first presentation to the hospital, these markers either accurately identify ALI at a time when current markers are normal or have prognostic utility once ALI is established, such as hyperacetylated HMGB1^5^ and blood lactate concentration.^26^ The present study adds a novel, translational biomarker of kidney injury into this panel, and further clinical development may result in prognostic models that are superior to current tools to aid management of patients and provide additional mechanistic insights into development of APAP‐induced hepatotoxicity.

Author names in bold designate shared co‐first authorship.
